# Heterogeneity and Dynamics of Vasculature in the Endocrine System During Aging and Disease

**DOI:** 10.3389/fphys.2021.624928

**Published:** 2021-03-09

**Authors:** Sina Stucker, Jessica De Angelis, Anjali P. Kusumbe

**Affiliations:** Tissue and Tumor Microenvironments Group, Kennedy Institute of Rheumatology, Nuffield Department of Orthopaedics, Rheumatology and Musculoskeletal Sciences (NDORMS), University of Oxford, Oxford, United Kingdom

**Keywords:** endocrine system, vascular niche, aging, vasculature, pancreas

## Abstract

The endocrine system consists of several highly vascularized glands that produce and secrete hormones to maintain body homeostasis and regulate a range of bodily functions and processes, including growth, metabolism and development. The dense and highly vascularized capillary network functions as the main transport system for hormones and regulatory factors to enable efficient endocrine function. The specialized capillary types provide the microenvironments to support stem and progenitor cells, by regulating their survival, maintenance and differentiation. Moreover, the vasculature interacts with endocrine cells supporting their endocrine function. However, the structure and niche function of vasculature in endocrine tissues remain poorly understood. Aging and endocrine disorders are associated with vascular perturbations. Understanding the cellular and molecular cues driving the disease, and age-related vascular perturbations hold potential to manage or even treat endocrine disorders and comorbidities associated with aging. This review aims to describe the structure and niche functions of the vasculature in various endocrine glands and define the vascular changes in aging and endocrine disorders.

## Introduction

The endocrine system is vital for efficient function and communication between different parts of the body and maintenance of homeostasis (Chrousos, 2007). It consists of various organs and glands including the gonads, pancreas and pituitary-, thyroid-, and adrenal- glands. Endocrine organs secrete signals, known as hormones, that are essential for maintaining the homeostasis. Many hormones in the body act on various organs, regulating a wide range of basic bodily functions. For instance, metabolic activities of muscle, adipose tissue, liver and other organs are regulated by insulin, adrenaline and noradrenaline. Sex development and characteristics are regulated by sex hormones such as testosterone and estrogen. Angiotensin and renin regulate blood pressure and renal filtration (Hiller-Sturmhöfel and Bartke, 1998; Kolka and Bergman, 2012; Maurer et al., 2016).

Hormones are transported via the bloodstream to reach their target tissues and cells. When binding to their target receptors, they induce an intracellular signaling cascade that triggers specific cellular responses.

The hypothalamus controls the production and secretion of numerous hormones in the pituitary gland via neuroendocrine signals. These pituitary hormones are subsequently released into the bloodstream and transported to target glands where they, in turn, trigger the release of hormones that affect organ function. Constant feedback mechanisms along this axis enable the regulation of hormone levels to maintain a stable system. Once hormones from target glands reach a certain concentration in the blood, this initiates a negative feedback loop that inhibits further hormone release in the hypothalamus and pituitary gland (Fliers et al., 2014; Keller-Wood, 2015; Ortiga-Carvalho et al., 2016). There are several regulatory hormonal cascades that rely on feedback mechanisms, including the hypothalamic-pituitary-thyroidal (HPT) or hypothalamic-pituitary-adrenal (HPA) axis (Hiller-Sturmhöfel and Bartke, 1998). Within these axes, hypothalamic releasing hormones such as thyrotropin-releasing hormone (TRH) and corticotrophin-releasing hormone (CRH) stimulate the pituitary gland to produce thyrotropin-stimulating hormone (TSH) and adrenocorticotrophic hormone (ACTH). TSH and ACTH then act on their respective target glands. TSH stimulates the release of thyroid hormones, while ACTH promotes adrenal cortisol production. Both cortisol and ACTH feedback on the hypothalamus and pituitary gland by inhibiting hypothalamic and pituitary hormone release and modulating pituitary and target gland sensitivity to hypothalamic releasing hormones and pituitary hormones, respectively (Hiller-Sturmhöfel and Bartke, 1998; Houshyar et al., 2001). In addition, increased plasma levels of corticosterone and ACTH reduce whole brain expression of glucocorticoid receptors (Houshyar et al., 2001).

The vasculature constitutes the primary transport system for hormones and is crucial for endocrine signaling. The endothelium poses a structural and functional barrier for hormone transport to their target cells. Specific changes in blood vessels (e.g., blood volume and pressure) can affect the release of certain hormones that, themselves can also modulate the endothelium and its function, for instance via controlling the production of growth factors and other hormones that regulate angiogenesis (Hiller-Sturmhöfel and Bartke, 1998; Clapp et al., 2009; Kolka and Bergman, 2012). Vasoactive hormones such as insulin (Tack et al., 1996), estradiol (Gilligan et al., 1994) and testosterone (Yue et al., 1995) are able to modulate the vessel diameter by inducing vasodilation or -constriction, thereby modulating the vascular surface area for exchange (Kolka and Bergman, 2012). The endothelium itself possesses an endocrine function and is often considered as part of the endocrine system. For instance, endothelial cells (EC) release various vasoactive signals such as a nitric oxide (NO) that cause vasorelaxation or vasoconstriction (Henderson and Henderson, 1995).

Most hormones are released in pulses that cause rapid or episodic increase in circulating concentrations. This is important for regulation of target cell function. This pulsatile pattern of hormone release relies on tight temporal control of hormone secretion and entry into the bloodstream (Marie et al., 2011). This is achieved by a complex interplay between the endothelium and endocrine cell that ensures precise temporal uptake and transport of hormones by the blood vessels (Marie et al., 2011). However, the structure and function of the microvasculature in many endocrine glands remain poorly understood. Therefore, this review aims to describe the structure and function of blood vessels in different endocrine glands. Secondly, this review will define vascular perturbations in aging and various endocrine disorders.

## Anatomy, Structure, and Heterogeneity of Blood Vessels in the Endocrine Glands

Endocrine glands are typically supplied by larger vessels that give rise to a dense network of capillaries. This microvascular network enables close interaction between endocrine cells and the vasculature (Henderson and Moss, 1985; Augustin and Koh, 2017). The specialized microvascular endothelium of endocrine glands is highly permeable to allow rapid hormone release and response to changes in homeostasis (LeCouter et al., 2001). Several endocrine glands, including thyroid and pituitary gland, contain fenestrated capillaries with intracellular pores of varying permeability that enable the exchange of nutrients, hormones and small peptides. Sinusoidal capillaries have larger gaps between ECs that enable the free exchange of water, plasma proteins and other larger solutes. In sinusoidal vessels, the blood flow decelerates to prolong the time of exchange between blood and interstitial fluid (Augustin and Koh, 2017).

Despite advances in understanding the specialization of the vasculature in organs such as liver and bone (Ding et al., 2014; Kusumbe et al., 2014, 2016; Augustin and Koh, 2017), the specialized structure and function of the vascular system in endocrine organs remains elusive. In the following paragraphs, we will briefly describe the anatomical structure of various endocrine glands, including afferent and efferent large vessels and the small capillaries.

### Testis

The testes are part of the male reproductive system. Their main functions include testosterone production and spermatogenesis, which is essential for male fertility. Among other functions, testosterone regulates testicular blood flow and vasomotion (Damber et al., 1992; Collin et al., 1993). The testis is comprised of multiple lobules containing two distinct compartments that are closely interconnected. The interstitial compartment that makes up around 15% of the human testicular volume contains Leydig cells (LCs) that are the main source of testosterone (Maddocks and Setchell, 1988; Ilacqua et al., 2018). The avascular tubular compartment, comprised of convoluted seminiferous tubules, occupies approximately 60-80% of the total testicular volume in humans and is the location of spermatogenesis (Ilacqua et al., 2018). In these tubules, nutrients are transported via the interstitial fluid, the formation of which is regulated by interstitial vessel permeability (Sharpe, 1983; Park et al., 2018). Also, the tubular compartment also contains germ cells and Sertoli cells (SCs) that reside in the basal membrane, extending into the lumen of the seminiferous tubuli. SCs promote germ cell maturation and adult sperm production and form the blood-testis barrier via expression of specialized tight junctional molecules (Ilacqua et al., 2018).

Testicular blood supply is provided via the testicular artery that originates from the abdominal aorta. Each lobule is supplied with blood via one main artery that branches into an elaborate bed of intratesticular arteries and capillaries between the seminiferous tubules. Testicular microvasculature is closely linked to seminiferous tubules and interstitial clusters of LCs (Ergün et al., 1994). Arterioles are enwrapped by LCs and branch into capillaries that innervate the wall of the seminiferous tubules, adapting to the coiling of the tubules (Ergün et al., 1994). Upon leaving the tubular wall, capillaries continue as post-capillary venules that enter an intricate network of veins wrapped around the testicular artery. This intertubular capillary network unites into the testicular vein. The testicular vein leaves the testis, draining into the inferior vena cava and the renal vein (Harrison and Barclay, 1948; Lupiáñz et al., 2012).

The major functions of the testicular vasculature include the regulation of testicular temperature and the transport of nutrients, metabolites and hormones. It transports pituitary gonadotropins to promote testicular spermatogenesis and testosterone production. Conversely, testosterone is transported to various target tissues throughout the body (Lupiáñz et al., 2012; Ilacqua et al., 2018). Moreover, testicular hormones regulate hypothalamic and pituitary output in classically defined feedback mechanisms (Matsumoto and Bremner, 1987; Roser, 2008).

In mammals, testicular microvessels are locally regulated via vasomotion, which is important for testicular function by affecting blood flow, transvascular fluid exchange and interstitial fluid formation (Collin et al., 2000; Lysiak et al., 2000). In combination with the high oxygen consumption due to spermatogenesis demands, the testicular environment contains low oxygen levels. In line with this, rat and mouse testis show constitutive expression of the transcription factor hypoxia-induced factor-1 (HIF-1) that is stabilized under hypoxic conditions and regulates oxygen homeostasis (Powell et al., 2002; Lysiak et al., 2009; Colli et al., 2019). Hypertension has been shown to impair testicular vasomotion, alter vascular morphology and increase HIF-1 expression in rats, suggesting a drop of oxygen levels in hypertensive rat testes (Colli et al., 2019). Moreover, hypertensive rats showed increased vascular endothelial growth factor (VEGF) levels and decreased sperm concentration and quality, indicating an essential role for blood pressure and vasomotion in testicular function (Colli et al., 2019). Additionally, ECs are essential for maintaining the spermatogonial stem cell (SSC) niche, with testicular endothelial cells expressing organ specific growth factors that are essential for maintaining SSC self-renewal. Disruption of key signaling pathways of testicular endothelial cells, such as in down syndrome, can lead to reduced fertility (Bhang et al., 2018).

### Ovary

The ovaries are the female gonads located on either side of the uterus. Anatomically, the ovary can be divided into three zones, the cortex, medulla and hilus. The blood supply in ovaries is provided via the ovarian artery that anastomoses with a branch of the uterine artery. The ovarian artery splits into smaller arterial branches that penetrate the hilus and medulla. Medullary arteries and arterioles show pronounced coiling and branching and form a plexus from which smaller arterioles originate that penetrate the cortex, forming a dense and highly fenestrated vascular network. Ovarian arteries and arterioles are accompanied by veins that merge into the ovarian vein at the hilus. The left ovarian vein drains into the renal vein, and the right ovarian vein drains into the vena cava (Clement, 1987; Kozik, 2000).

Anatomically, the ovary contains a large number of growing follicles in the cortex and medulla that modulate the vasculature according to their changing needs during follicular development (Brown and Russell, 2014). Within each follicle, angiogenesis is regulated independently, forming an individual capillary network (Fraser, 2006). Compared to the relative quiescent nature of the vascular system in the adult, the follicular vasculature is remarkably active, exhibiting dynamic changes in angiogenesis, vascular permeability and blood flow during different stages of the ovarian cycle. Before ovulation, the dominant follicle exhibits increased blow flow and follicular size (Acosta et al., 2003), whereas angiogenesis and vascularity peaks during the formation of the corpus luteum (CL) after ovulation (Brown and Russell, 2014). This continuous cyclic remodeling of the vascular system is crucial for follicular and luteal development and normal ovarian function (Augustin et al., 1995; Brown and Russell, 2014).

Four-dimensional time-lapse imaging of gonad vascularization shows a sex-specific pattern of gonadal vasculature. In the XY gonad, mesonephric blood vessels break down and release mesonephric ECs that migrate into the developing testis to form the major testicular artery. These mechanisms correlate with a rapid morphogenesis and change in direction of testicular blood flow and may increase testicular blood flow to enhance testosterone export during secondary sex determination (Brennan et al., 2002; Coveney et al., 2008). In contrast, the ovary is relatively quiescent. The ovarian vasculature grows from pre-existing vessels independently of mesonephric vasculature (Brennan et al., 2002; Coveney et al., 2008). VEGFA-VEGFR2 signaling plays an important role in gonadal morphogenesis and vasculogenesis and angiogenesis, promoting EC survival, differentiation and migration (Bott et al., 2006, 2010). In the ovary, VEGFA is expressed in granulosa and theca cells in ovarian follicles, and pharmacological inhibition of VEGFA signaling drastically reduces ovarian vascular density by 94% and disrupts follicular development (McFee et al., 2009). Similar experiments in rat testis demonstrate VEGFA expression in SCs. Here, inhibition of VEGFA signaling results in a 90% reduction of vascular density and inhibition of seminiferous tube formation *in vitro* (Bott et al., 2006). Collectively, these studies highlight the importance of VEFA in gonadal morphogenesis and vascularization. During fertility treatment, the ovaries can respond to Human Chorionic Gonadotropin to upregulate VEGF, increasing vascular permeability in ECs, leading to Ovarian hyperstimulation syndrome (Albert et al., 2002; Fang et al., 2020).

### Thyroid Gland

The thyroid gland is one of the largest endocrine glands in the human body and resides in the lower neck, anterolaterally to the trachea and larynx. It is composed of a left and a right lobe interconnected by an isthmus (Ozgur et al., 2011; Policeni et al., 2012). Blood supply to the thyroid gland is provided by two pairs of inferior and superior thyroid arteries that branch from the thyrocervical arteries and the external carotid arteries, respectively (Loevner, 1996). These inferior and superior thyroid arteries have many anastomoses, creating a rich basket-like capillary network around thyroid follicles (Fujita and Murakami, 1974; Cozzolino et al., 2005). The venous system is formed by a venal plexus that drains blood into the internal jugular vein (via the superior and middle thyroid veins) and the brachiocephalic vein (via the inferior thyroid vein) (Loevner, 1996; Policeni et al., 2012).

Thyroid microvasculature is heavily fenestrated with distinct clusters of fenestrations and depends on VEGF signaling. Inhibition of VEGF via administration of AG013736, a small molecule inhibitor of VEGFRs drastically reduced both capillary vascularity and fenestrations in adult mouse thyroids (Inai et al., 2004; Kamba et al., 2006). Furthermore, thyroid capillaries are supported pericytes that express NG2 or PDGFRβ and consistently wrap along the length of capillaries (Kamba et al., 2006).

The core function of the thyroid gland is the production of essential thyroid hormones, including triiodothyronine (T_3_) and thyroxine (T_4_), that are important in metabolic processes. Thyroid hormone secretion is mediated via feedback mechanisms along the hypothalamic-pituitary axis. TRH from the hypothalamus stimulates the release of a TSH by the pituitary that acts on the thyroid gland, promoting the thyroid hormone secretion (Loevner, 1996; Policeni et al., 2012). In the bloodstream, T_3_ and T_4_ are transported in their form by carrier proteins such as thyroxine-binding globulin and albumin. Only small fractions of of T_3_ and T_4_ exist in an unbound, active form. While T_4_ is produced entirely in the thyroid gland, only a small proportion of T_3_ is synthesized here, whereby the majority of T_3_ synthesis takes place peripherally via conversion of T_4_ (Loevner, 1996; Vita et al., 2019).

### Pituitary Gland

The pituitary gland, also called hypophysis, is an endocrine gland attached at the base of the hypothalamus. Despite its small size of approximately 10mm, it is essential to maintain homeostasis and hormonal balance and functions as the central endocrine regulator. Anatomically, the pituitary gland consists of two compartments that act as independent endocrine organs with distinct cytology, outputs and regulation (Amar and Weiss, 2003). The adenohypophysis, composed of epithelial cells, consists of the anterior lobe and the pituitary stalk or infundibulum that connects the pituitary gland to the brain. The neurohypophysis describes the posterior lobe that is derived from neural ectoderm. The anterior and posterior lobes are is connected via the pars intermedia (Amar and Weiss, 2003).

The adenohypophysis contains acini with five types of endocrine cells, including corticotropic, somatotropic, mammotropic, gonadotropic and thyrotropic cells that produce ACTH, growth hormone (GH), prolactin (PRL), luteinizing hormone (LH) and follicle-stimulating hormone (FSH), and TSH, respectively (Larkin and Ansorge, 2000). Although most pituitary acini contain a mixture of these endocrine cell types, cellular distribution is not random. While acini in the lateral lobe contain mostly somatotrophs and lactotrophs, corticotrophs are located primarily in the center of the adenohypophysis (Larkin and Ansorge, 2000). The center of the acini is occupied by non-hormone producing follicular-stellate (FS) cells that have extended processes between the endocrine cells and are thought to act as stem cells that give rise to endocrine cells (Horvath and Kovacs, 2002).

The adenohypophysis is considered the most highly vascularized mammalian tissue and is mainly supplied by a set of superior hypophyseal arteries (SHAs) that originate from the internal carotid artery (Page, 1982; Amar and Weiss, 2003). The SHA branches into smaller arteries that anastomose with branches from the contralateral SHA, forming a rich primary plexus of fenestrated capillaries at the top of the pituitary stalk. The fenestrated capillaries merge into venules that subsequently drain into larger portal veins that advance into the anterior lobe to form a secondary plexus. This secondary plexus then drains into efferent lateral hypophyseal veins (Daniel, 1966). Adenohypophyseal hormones in the second plexus can also reflux to the primary plexus to modulate their own synthesis via feedback mechanisms (Page, 1982; Amar and Weiss, 2003).

The neurohypophysis exhibits a very different histology compared to the nested organization of endocrine cells in the adenohypophysis. Instead, it contains axons from hypothalamic neurons, forming a hypothalamic-hypophyseal tract. These axon terminals release their neurosecretory products, including oxytocin and vasopressin and are surrounded by elongated pituicytes (Larkin and Ansorge, 2000; Le Tissier et al., 2017). The neurohypophysis is supplied by a set of inferior hypophyseal arteries (IHAs) that divide into ascending and descending branches that anastomose with the branches on the contralateral side, forming an arterial ring that receives neurosecretory products from the axon terminals (Page, 1982; Lechan and Toni, 2000; Amar and Weiss, 2003).

### Adrenal Gland

The adrenal glands are in the retroperitoneum, located above the kidneys. Through the production of two major types of hormones, catecholamines and steroids, they are an important regulator of metabolic, immune and cardiovascular processes. The adrenal gland can be divided into the cortex and medulla which have distinct histology and function.

The adrenal cortex contains adrenocortical cells that are organized into three subzones (zona glomerulosa, zona fasciculata, zona reticularis). These cortical subzones exhibit characteristic histology and secrete different steroid hormones (Idelman, 1970; Miller and Auchus, 2011; Sun et al., 2018). The zona glomerulosa produces mineralocorticoids that are involved in the regulation of blood pressure and electrolyte balance. Endocrine cells in the zona fasciculata are the source of glucocorticoids which play an important role in metabolism and immune response. Glucocorticoid secretion is regulated by the hypothalamic-pituitary-adrenal (HPA) axis that includes hypothalamic CRH. CRH stimulates pituitary corticotropes to secrete ACTH that ultimately regulates adrenal steroidogenesis. The zona reticularis secretes androgens. In contrast, the adrenal medulla produces catecholamine hormones, including epinephrine, also known as adrenaline, and norepinephrine that drive the stress response (Bassett and West, 1997; Vinson, 2016; Sun et al., 2018).

The left and right adrenal glands are supplied by the left renal artery and the inferior phrenic artery, respectively. These arteries form an arteriolar plexus that branches into smaller vessels, innervating the medulla and draining into a central vein (Murakami et al., 1989; Bassett and West, 1997). The cortex is supplied by vessels that arise from the capsular plexus and anastomose in the zona glomerulosa. This anastomotic network flows into sinusoidal capillaries that continue through the zona fasciculata before draining into larger sinusoids in the zona reticularis that run into the central vein (Idelman, 1970; Vinson et al., 1985; Bassett and West, 1997).

### Pancreas

The pancreatic gland contains small lobules, containing both endocrine and exocrine tissue and plays a crucial role in digestion and glucose homeostasis. The majority of pancreatic cells are exocrine and are organized in acini that release bicarbonate and digestive enzymes into the duodenum. Endocrine cells makeup only 1-2% of the pancreatic tissue and are clustered in islets of Langerhans scattered throughout the exocrine tissue (In’t Veld and Marichal, 2010). Endocrine islet cells can be divided into subtypes including α-, β-, δ-, and PP-cells that secrete distinct metabolic-regulating hormones including glucagon, insulin, somatostatin and pancreatic peptide, respectively (Ballian and Brunicardi, 2007).

The islets of Langerhans are embedded within a dense network of specialized microvessels that is distinct from that of the exocrine pancreas (Goldstein and Davis, 1968; Ballian and Brunicardi, 2007; Olerud et al., 2009). The vascular system in these islets is crucial for islet function and intercellular communication between endocrine and exocrine cells (Ballian and Brunicardi, 2007) and is supported by NG2 or PDGFRβ-expressing pericytes (Kamba et al., 2006). The capillary network in pancreatic islets highly expresses VEGFR2 and VEGFR3 and is dependent on VEGF signaling. Inhibiting VEGF signaling leads to a drastic vascular regression of islet capillaries in adult mice (Kamba et al., 2006). This capillary loss can also be observed in multiple other endocrine glands, including the adrenal, pituitary and thyroid glands. In contrast, the vascular density of many other organs such as heart, brain, retina, lung and skeletal muscle did not show significant changes. These findings indicate a large proportion of VEGF-vascular beds in the endocrine system of adult mice (Kamba et al., 2006).

Under basal conditions, islets are hyperperfused, enabling adjustment to insulin secretion under constant blood flow (Johansson et al., 2005). Each islet is innervated by one to five afferent arterioles that branch into a network of capillaries surrounding endocrine cells to enable a sufficient supply of oxygen and nutrients (Brunicardi et al., 1996). Depending on islet size, venous blood exits either directly into veins or drains into the insulo-portal system to perfuse exocrine pancreatic tissue. In turn, the exocrine tissue can also deliver blood to islets, indicating a bilateral communication between endocrine and exocrine pancreatic tissue (Murakami et al., 1992; Ballian and Brunicardi, 2007).

In rodents, islets show a topographical cytoarchitecture and microcirculation. Blood flows to the islet core, where mostly β-cells reside and exits through venules in the periphery (Murakami et al., 1993; Ballian and Brunicardi, 2007). The human islets of Langerhans, however, do not show a cellular topography. Instead, α-, β-, and δ- cells are scattered throughout the islets without significant clustering (Cabrera et al., 2006). In both humans and rodents, the majority of β-cells are aligned along capillaries, and vascular cells and are organized in a ‘rosette-like’ structure (Bonner-Weir, 1988; Cabrera et al., 2006; Bonner-Weir et al., 2015).

Furthermore, β-cells exhibit a high degree of phenotypic and functional heterogeneity with multiple studies reporting variations in size, granularity, membrane potential, glucose responsiveness and, insulin secretion (Dean and Matthews, 1968; Cabrera et al., 2006; Wojtusciszyn et al., 2008; Katsuta et al., 2012; Roscioni et al., 2016). This β-cell heterogeneity depends on differences in the pancreatic microenvironment that is created, in part, by distinct islet vascularization and blood perfusion patterns (Ellenbroek et al., 2013). Whole-mount imaging and three-dimensional analysis of islet vascular architecture demonstrate changes in vascularization depending on size and location within the pancreas. For example, larger islets have more vascular penetration points than smaller islets, and central islets are supplied by larger vessels, while peripheral islets may receive capillaries in a polarized fashion (El-Gohary et al., 2012; Roscioni et al., 2016). Likewise, islets also differ in blood perfusion and can be divided into low-blood perfused islets with low oxygen consumption and high blood perfused islets with high oxygen consumption. Multiple *in vivo* studies demonstrate increased β-cell proliferation, insulin secretion and stress susceptibility in high-blood compared to low-blood perfused islets (Olsson and Carlsson, 2011; Lau et al., 2012; Ullsten et al., 2015). ECs from pancreatic islets bidirectionally communicate with β-cells to increase glucose medicated insulin secretion (Johansson et al., 2009). Changes in islet vasculature can influence β-cell mass and are linked with diabetes (Staels et al., 2019).

Alonside vasculature, islets are innervated by the autonomic nervous system that controls islets architecture and maturation. Of interest, genetically or pharmacologically induced ablation of the sympathetic nerve fibers in mice, significantly alters islet architecture and impairs insulin secretion and glucose tolerance (Borden et al., 2013). In contrast to murine islets, human islets are sparsely innervated by autonomic axons, suggesting an indirect regulation of hormone secretion by via sympathetic control of local blood (Rodriguez-Diaz et al., 2011).

## Niche Functions of Blood Vessels in the Endocrine System

The microvascular blood vessel network plays an essential role in tissue development and function via its ability to transport nutrients and oxygen to all tissues throughout the body. The local microvasculature in endocrine glands interacts with local endocrine cells to maintain their function and homeostasis (Colin et al., 2013). ECs achieve this through the secretion of a variety of paracrine factors such as growth factors and cytokines, collectively termed angiocrine signals. Angiocrine signals are crucial for stem and progenitor cell maintenance, differentiation, and function (Colmone and Sipkins, 2008).

This vascular microenvironment is also referred to as vascular niche, and has been described in different organs and tissues. Vascular microenvironments are involved in a wide range of physiological and pathological processes (Augustin and Koh, 2017). In the bone marrow, ECs have been identified as a critical component in the maintenance of the hematopoietic stem cell (HSC) niche (Hooper et al., 2009; Morrison and Scadden, 2014). Here, ECs show a striking morphological and functional diversity and growing evidence suggests an organotypic function of endothelium that regulates development and homeostasis. This diversity enables them to adapt to local needs and supports distinct tissue-specific functions (LeCouter et al., 2001; Cleaver and Melton, 2003; Nolan et al., 2013). However, the role of vascular niches in the endocrine system remains incompletely understood. In the following section, we will describe the niche functions of the vasculature in different endocrine glands (Table 1).

**TABLE 1:** Vascular niche associated factors in the endocrine system in homeostasis, aging, and endocrine disorders.

Sl. No	Factor/Signal	Function	Cell Type	Condition	References
1	Angiopoietin-1	Angiogenesis, ovarian follicular development, ovulation	ECs, follicular cells		Xu and Stouffer, 2005; Abramovich et al., 2009
2	Angiotensin-1	Aldosterone release	Adrenocortical cells		Rosolowsky and Campbell, 1994; Ansurudeen et al., 2006
3	CSFR1	SSC renewal	SSC		Oatley et al., 2009
4	EG-VEGF	EC proliferation, migration and fenestration	ECs		LeCouter et al., 2001
5	Endothelin	Aldosterone and corticosterone release, insulin secretion	Adrenocortical cells, β-cells		Rosolowsky and Campbell, 1994; Belloni et al., 1996; Gregersen et al., 1996
6	Ezh2	β-cell expansion	β-cells	Aging	Chen et al., 2011
7	FGF-2	Mediation of endothelial GDNF, corpus luteum vascularization	ECs		Berisha et al., 2000; Bhang et al., 2018
8	Fibronectin, laminin, collagen	β-cell proliferation, insulin gene expression	β-cells		Kaido et al., 2004; Nikolova et al., 2006
9	GDNF	SSC survival and maintenance	SSCs		Kubota et al., 2004; Bhang et al., 2018
10	Gja1	Pancreatic islet capillary maintenance	ECs	Aging	Chen et al., 2020b
11	Glucocorticoids	Inhibition of angiogenesis, TSP-1 production	ECs	Aging	Logie et al., 2010; Yang et al., 2018
12	HIF-1α	Angiogenesis, VEGFA secretion, oxygen homeostasis	ECs	Hypertension	Gérard et al., 2009; Lysiak et al., 2009; Colli et al., 2019
13	HGF	β-cell proliferation	β-cells		Johansson et al., 2006
14	ICAM-1	Inflammation, immune cell recruitment	Immune cells	Aging	Chen et al., 2020b
15	Jagged1	Pituitary stem cell maintenance	Pituitary stem cells		Tando et al., 2013
16	MMPs	Periovulatory basement membrane breakdown, EC invasion, ECM remodeling, fibrosis	ECM	Aging, diabetes	Kano et al., 2005; Su et al., 2008; Almaça et al., 2014
17	Nitric oxide	Vasodilation, corticosterone and aldosterone production	ECs, adrenocortical cells	Diabetes, aging	Marin et al., 1999; Romero Maritza et al., 2008
18	NOTCH2	Perivascular progenitor maintenance	Perivascular progenitor cells		Kumar and DeFalco, 2018
19	p53	Insulin resistance, inflammation, lypolysis	Adipocytes	Obesity	Minamino et al., 2009; Vergoni et al., 2016
20	PDGFβ	EC proliferation and migration, pericyte activation, β-cell maintenance	ECs, pericytes, β-cells	PCOS	Nilsson et al., 2006; Woad et al., 2009; Chen et al., 2020b
21	Smad	Thyroid development, EC differentiation	Thyrocytes, ECs		Hick et al., 2013; Villacorte et al., 2016
22	TGF-β1	β-cell maintenance, angiogenesis	β-cells, follicular cells	PCOS	Tal et al., 2013; Liu et al., 2015; Jiang et al., 2018
23	Thy-1	Ovarian follicular growth, cellular differentiation	Granulosa cells		Bukovský et al., 1995
24	TSP-1	Inhibition of angiogenesis, TGF-β1 activation	ECs	PCOS	Crawford et al., 1998; Thomas et al., 2008; Liu et al., 2015
25	VEGFA	Angiogenesis, ovarian follicular growth, corpus luteum vascularization, seminiferous tube formation	ECs	Aging	Ferrara et al., 1998; Berisha et al., 2000; Klein et al., 2000; Bott et al., 2006; Yang and Fortune, 2007; McFee et al., 2009
26	VEGFR2	Fenestrae formation, follicular cell proliferation	ECs, follicular cells		Jang et al., 2017

### Angiocrine Factors in Testis

In the testis, the convoluted seminiferous tubules are surrounded by interstitial tissue that contains blood vessels, LCs and other perivascular cells. The basal compartment of the seminiferous tubules contains spermatogonia in various stages of differentiation, including spermatogonial stem cells (SSCs) that are crucial for spermatogenesis and fertility (Desjardins and Ewing, 1993; Russell et al., 1993; Ogawa et al., 2005). These SSCs reside in a specialized stem cell niche that is, at least partially, maintained by testicular endothelial cells (TECs). TECs produce several factors to support SSCs survival and maintenance, including glial cell line-derived neurotrophic factor (GDNF) (Kubota et al., 2004; Bhang et al., 2018). Endothelial GDNF production is mediated via fibroblast growth factor 2 (FGF-2) and fibroblast growth factor receptor 1 (FGFR1) signaling that activates the calcineurin pathway. Transplantation of TECs in chemotherapy-treated mice restored spermatogenesis, demonstrating an important role for TECs in SSC self-renewal and testicular regeneration (Bhang et al., 2018).

**FIGURE 1:**
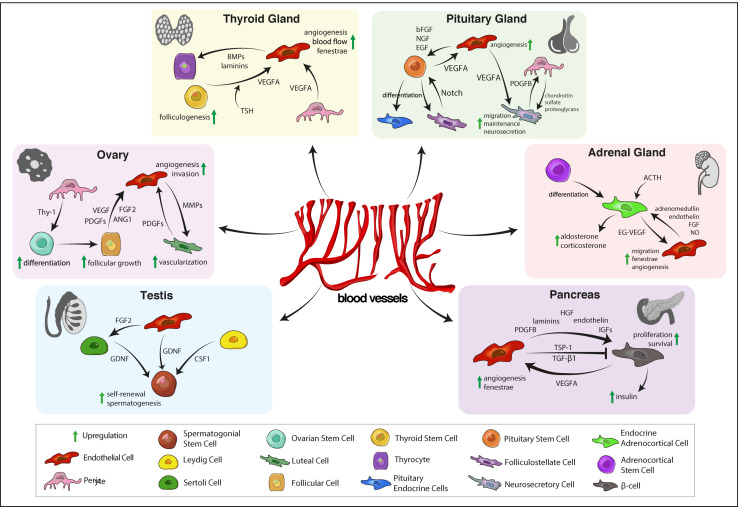
Vascular niche functions in the endocrine system. In the testis, ECs release various endocrine signals to maintain SSCs and spermatogenesis. OSC maintenance is supported by pericytes. During follicular and luteal stages of the cycle, growth factors regulate periodic growth and regression of ovarian vasculature that is needed for follicular and luteal development. In the thyroid, angiogenic signals from TSCs and pericytes regulate angiogenesis, endothelial fenestrae formation that is important for thyrocyte function. Pituitary ECs and pericytes promote maintenance and function of neurosecretory cells in the neurohypohysis and pituitary stem cells in the adenohypophysis. Angiocrine signals also regulate endocrine function of the adrenal cortex, that, in turn, promotes angiogenesis via the endocrine gland-specific growth factor EG-VEGF. In the pancreas, reciprocal interaction between ECs and β-cells is required for angiogenesis and insulin secretion. EC, endothelial cell; SSC, spermatogonial stem cell; FGF2, fibroblast growth factor 2; GDNF, glial cell line-derived neurotrophic factor; CSF-1, colony-stimulating factor 1; OSC, ovarian stem cell; PDGF, platelet-derived growth factor, VEGF, vascular endothelial growth factor; ANG1, angiopoietin 1; MMP, matrix metalloproteinase; TSC, thyroid stem cell; TSH, thyrotropin-releasing hormone; BMP, bone morphogenetic protein; bFGF, basic fibroblast growth factor; NGF, nerve growth factor; EGF, epidermal growth factor; EG-VEGF, endocrine gland-derived vascular endothelial growth factor; NO, nitric oxide; HGF, hepatocyte growth factor; IGF, insulin-like growth factor; TSP-1, thrombospondin-1; TGF-β1, transforming growth factor β1.

LCs contribute to SSC maintenance by expression colony-stimulating factor 1 receptor (CSF1R) that promotes SSC self-renewal (Oatley et al., 2009; Figure 1). Time-lapse imaging of GFP-labeled undifferentiated spermatogonia demonstrates a preferential localization of undifferentiated spermatogonia near intertubular vessels and interstitial LCs (Yoshida et al., 2007). Upon differentiation, spermatogonia move away from intertubular vessels, dispersing throughout the basal compartment of the seminiferous tubules. This relocation of spermatgonia is accompanied by a vascular reorganization. Transplantation of seminiferous tubules triggers the formation of vasculature with SSCs localizing along with the newly established vascular pattern in the graft (Yoshida et al., 2007; Yoshida, 2018). This demonstrates a crucial role for interstitial cells and vessels in SSC maintenance and stem cell niche establishment.

Within the seminiferous tubules, somatic SCs also express GDNF and have been implicated in SSC niche formation. SSC transplantation into host mice with polythiouracil (PTU)-induced increase in SC numbers enhanced vascular niches. Transplanting SSCs from PTU-treated donors into normal recipients significantly increased SSCs numbers by more than 3-fold, indicating a key role for SCs in regulating SSC and niche abundance (Oatley et al., 2011).

Fetal testis provides a perivascular microenvironment for multipotent progenitor cells (Kumar and DeFalco, 2018). These perivascular multipotent progenitor cells are Notch-active and Nestin-positive and give rise to several interstitial cell types, including LCs, pericytes and smooth muscle cells. Vascular inhibition disrupts Notch signaling in these progenitors, stimulating excessive LC differentiation. Thus, angiocrine Notch signals crucially regulate the balance of LC differentiation, highlighting the importance of the vasculature for interstitial progenitor cell maintenance (Kumar and DeFalco, 2018).

### Vascular Microenvironments in Ovary

Ovarian function depends on the periodic growth regression of the ovarian vasculature and variations in blood flow rate. Ovarian vasculature provides blood and nutrients to follicles and the CL and regulates steroid production. Small follicles are avascular and rely on the stromal vasculature (Mariana Di et al., 2018). Beyond the primary stage, follicle growth requires the formation of an individual capillary network in the thecal layer of each follicle. Vessel formation and regression are mediated via angiogenic factors such as VEGFA, platelet-derived growth factor (PDGF), angiopoietins (Angs) and thrombospondin-1 (TSP-1) that stimulate EC proliferation, migration, and vascular stability (Tamanini and De Ambrogi, 2004; Nilsson et al., 2006; Yang and Fortune, 2007; Abramovich et al., 2009; Figure 1). Multiple studies demonstrate that inhibition of angiogenesis via blockade of VEGFA signaling or administration of antiangiogenic compounds, disrupts follicular growth and ovulation, and completely inhibits CL vascularization (Ferrara et al., 1998; Wulff et al., 2002; Kuhnert et al., 2008; Robinson et al., 2009). Preovulatory follicles show an increased Ang1:Ang2 ratio (Hayashi et al., 2004) and Ang2 injection into monkey follicles delayed follicle maturation and inhibited ovulation by disrupting EC-pericyte interactions (Xu and Stouffer, 2005). Perivascular cells in the endocrine system can be marked by perivascular markers such as platelet-derived growth factor receptor β (PDGFRβ), NG2 and α-SMA. A recent deep imaging study by Chen et al. (2020b) visualized PDGFRβ and NG2 and α-SMA expressing perivascular cells in multiple glands of the endocrine system in both rodents and humans.

The antiangiogenic factor TSP-1 is upregulated during follicular atresia in marmoset monkeys and has been suggested to play an important role in follicular breakdown via the inhibition of angiogenesis (Thomas et al., 2008).

During ovulation, the basement membrane is broken down, enabling EC and pericyte invasion and rapid vascularization of the developing CL. This vascularization is likely mediated by VEGFA and FGF2 that accumulates before ovulation in the later stages of follicular development (Berisha et al., 2000; Robinson et al., 2009). Periovulatory breakdown of the basement membrane requires proteases that degrade components of the extracellular matrix (ECM). In line with this, the metalloproteinase ADAMTS1 is transiently upregulated in ECs (Su et al., 2008; Robinson et al., 2009). Matrix metalloproteinases (MMPs) are also produced by pericytes that are activated by luteal PDGF-signaling (Kano et al., 2005; Robinson et al., 2009; Woad et al., 2009).

ECs and pericytes also play an important role in the maintenance of ovarian stem cells (OSCs). In adult ovaries, OSCs give rise to germ and granulosa cells and reside in a stem cell niche in the ovarian surface epithelium (Bukovsky et al., 2004; Flesken-Nikitin et al., 2013). Within this niche, vascular pericytes facilitate the formation of secondary germ cells. These germ cells migrate towards cortical vessels that transport them to granulosa cell nests in the lower cortex to form primordial follicles (Bukovsky, 2011). In addition, pericytes release the morphoregulatory Thy-1 differentiation protein, that is associated with cellular differentiation and macrophage presence. Thy-1 is released among granulosa cells to initiate the growth of resting follicles (Bukovský et al., 1995; Bukovsky, 2011).

### Vascular Niches in Thyroid Gland

In the thyroid gland, follicular cells and surrounding capillaries form an angiofollicular unit to control endocrine thyroid function (Gérard et al., 2002; Colin et al., 2013). Independent of TSH stimulation, angiofollicular units can induce microvascular responses to preserve thyroid hormone synthesis. For instance, when intracellular iodine levels drop, follicular cells increase HIF-1α expression, which is accompanied by an increase in ROS generation, stabilizing HIF-1α. The subsequent increase of follicular VEGFA secretion activates neighboring ECs and pericytes, resulting in microvascular expansion and elevated blood flow (Gérard et al., 2009; Colin et al., 2013). Furthermore, genetic depletion of VEGFR2 and pharmacological blockade of VEGFA or VEGFR2 in mice demonstrates that endothelial VEGFA-VEGFR2 signaling promotes fenestrae formation and follicular cell proliferation (Jang et al., 2017; Figure 1).

In adult thyroids, thyroid stem cells constitute a very small proportion of thyroid cells. They maintain their proliferation and differentiation ability, enabling regeneration of thyrocytes and reconstruction of thyroid follicles (Thomas et al., 2006; Lan et al., 2007; Gibelli et al., 2009). Folliculogenesis requires the formation of an epithelial basement membrane which is controlled by bone morphogenetic protein (BMP)-Smad signaling in thyrocytes and EC invasion into the developing thyroid (Hick et al., 2013; Villacorte et al., 2016). BMP-Smad signaling also regulates VEGFA expression in thyroid progenitors. Thyroid-specific double knockout of Smad1 and Smad5 impairs thyroid development, follicle architecture, endothelial differentiation and basement membrane assembly. Conditioned medium from embryonic endothelial progenitor cells (eEPCs) rescues the observed defects in folliculogenesis. Normal folliculogenesis further requires laminins and type IV collagens that are produced by endothelial and epithelial components of the angiofollicular unit (Hick et al., 2013; Villacorte et al., 2016). These findings suggest a reciprocal communication between ECs and thyrocytes that creates a folliculogenic microenvironment.

### Vascular Microenvironments in Pituitary Gland

In the pituitary gland, hormone secretion is regulated by neuroendocrine signals from the hypothalamus and peripheral endocrine feedback mechanisms. The adult pituitary is able to adapt relative and absolute numbers of cells from its different endocrine cell types, enabling high adaptability of hormonal output/input responses to changing physiological demands (Levy, 2002; Fauquier et al., 2008). Therefore, adult adenohypophysis harbors various stem and progenitor cell populations. A small SOX2 and SOX9 expressing population of multipotent pituitary stem and progenitor cells gives rise to all pituitary endocrine cell types and parenchymal folliculostellate cells (Andoniadou et al., 2013; Rizzoti et al., 2013). In rodents, SOX2-positive pituitary stem and progenitor cells are mainly located in the marginal cell layer but can also be found in clusters scattered throughout the parenchyma of the adenohypophysis (Fauquier et al., 2008). A majority (85%) of SOX2-positive pituitary stem and progenitor cells express calcium-binding protein B (S100β) and produce various growth and angiogenic factors including VEGF (Ferrara et al., 1987; Gospodarowicz and Lau, 1989; Yoshida et al., 2011). S100β-positive cells in the marginal cell layer niche of transgenic S100β-GFP mice also express Notch receptors and ligands that are important for stem and progenitor cell maintenance (Tando et al., 2013).

Within these marginal cell layer and parenchymal niches, stem cells are regulated by growth factors such as basic fibroblast growth factor (bFGF), epidermal growth factor (EGF) and nerve growth factor (NGF) (Chen et al., 2006; Yoshida et al., 2016). Immunostaining for endothelial and stem cell markers further suggests the existence of a perivascular MSC population, as it has been shown in other organs such as the bone marrow (Garcia-Lavandeira et al., 2009, 2015; Méndez-Ferrer et al., 2010).

In the neurohypophysis, axon terminals store neurosecretory granules containing PDGF-β that may activate pericytes that highly express PDGFRβ. This may induce a shape conversion of these pericytes that can extend their processes to increase vascular protrusions, leading to a reorganization of the perivascular space in the neurohypophysis (Miyata, 2017; Nishikawa et al., 2017; Figure 1). Inhibition of VEGF-signaling decreased the density of neurosecretory axonal terminals and reduced the contact with the vasculature, indicating an important role in axonal maintenance. The neurohypophysis further shows an expression of chondroitin sulfate proteoglycans, that act as perivascular substrates for neuronal migration, indicating an important role for pericytes in support and migration of neural stem and progenitor cells (Morita et al., 2010; Furube et al., 2014).

### Blood Vessel-Derived Signals in Adrenal Gland

The adrenal medulla is the place of catecholamine production and mainly consists of chromaffin cells in close association with the medullary endothelium. Coculturing adrenal medulla-derived PC12 cells with bovine adrenal medullary ECs, stimulated chromaffin differentiation of PC12 cells, indicating an important role for ECs in organ-specific differentiation of the adrenal medulla (Mizrachi et al., 1990).

The adrenal cortex represents the site of steroid hormone production and contains various types of steroidogenic cells originating from self-renewing populations of undifferentiated somatic stem cell progenitors (Mitani et al., 2003). Ki67 labeling and BrdU pulse-chase labeling experiments show that these somatic stem cell progenitors are located in the outer cortex, where the majority of cell proliferation takes place. Subsequently, cells move inwardly along the cortical subzones towards the medulla (McNicol and Duffy, 1987; Chang et al., 2013). At the border to the medulla, they are eliminated by apoptosis (Zajicek et al., 1986; Carsia et al., 1996; Chang et al., 2013).

The dense fenestrated adrenocortical capillaries enable close contact of endocrine cells and ECs, which is important for paracrine signaling between vasculature and endocrine tissue (Thomas et al., 2003). Stimulation of human adrenocortical cells (in HUVEC conditioned medium) with angiotensin II or FSK significantly increases aldosterone release and sensitizes adrenocortical endocrine cells to angiotensin II stimulation (Ansurudeen et al., 2006). Since HUVEC conditioned medium contains a variety of angiocrine factors, the precise mechanisms of this effect remain largely unknown (Kanczkowski et al., 2017). Earlier studies demonstrate that EC-derived signals such as NO, endothelin and adrenomedullin stimulate aldosterone and corticosterone production in adrenocortical cells (Rosolowsky and Campbell, 1994; Belloni et al., 1996; Ehrhart-Bornstein et al., 1998; Rosolowsky et al., 1999). This stimulation may be mediated by NO-regulated activation of cyclic adenosine monophosphate (cAMP) (Ansurudeen et al., 2009). Another study suggests the involvement of a yet unknown protein of 3 KDa that may increase aldosterone secretion through activation of the protein kinase C (PKC) pathway (Rosolowsky and Campbell, 1994). Recently, endothelial FGF has also been associated with the β-catenin-induced adrenocortical activity (Schwafertz et al., 2017).

In turn, high levels of adrenocortical hormones such as glucocorticoids and catecholamines in the adrenal microenvironment can also influence vascular function. For instance, high plasma steroid levels have been shown to stimulate and sustain endothelial production of developmental endothelial locus-1 (Del-1), that is associated with adrenal inflammation (Kanczkowski et al., 2013).

Steroidogenic glands express a special endocrine gland-derived VEGF, termed EG-VEGF (LeCouter et al., 2001; Figure 1). Despite its structural distinction form VEGFA, EG-VEGF has a similar function. EG-VEGF induces proliferation, migration and fenestration in endocrine gland-derived ECs and promotes extensive angiogenesis while showing no effect on other ECs (e.g., HUVECs, human dermal microvascular cells, adult bovine aortic and bovine brain capillary cells) and non-EC types (pericytes, vascular smooth muscle cells, fibroblasts and keratinocytes), highlighting the tissue-specific regulation of vascular proliferation and differentiation (LeCouter et al., 2001).

### Vascular Niches in Pancreas

Endocrine pancreatic islets are vascularized by a dense and highly branched network of capillaries, whereas the surrounding tissue contains thinner quiescent capillaries (Zhou et al., 1996; Gorczyca et al., 2010). Islet ECs are characterized by distinct expression of cell surface markers that distinguishes them from the surrounding exocrine tissue (Yao et al., 2005). These markers include nephrin (Zanone et al., 2005), TSP-1, endostatin and the proteinase inhibitor alpha-1 that maintains their low proliferation rate (Lou et al., 1999; Cantaluppi et al., 2006; Mattsson et al., 2006). A recently identified subtype of islet capillaries is positive for CD31 and ESM-1 and shows high expression of Endomucin. This vessel subtype secretes growth factors involved in β-cell survival and maintenance, including Pdgfa, Pdgfb, Igf1, Igf2, Cxcl12 and stem cell factor (SCF) (Chen et al., 2020b).

ECs can directly affect β-cell function. For instance, islet capillaries can upregulate insulin secretion and promote β-cell survival via secretion of soluble factors and ECM proteins such as laminins, fibronectin and collagen in a β_1_-integrin-dependent manner (Kaido et al., 2004; Nikolova et al., 2006; Figure 1). Treatment of VEGF-A deficient mutant islets with vascular laminins rescued impaired β-cell proliferation and lead to an upregulation of insulin gene expression (Nikolova et al., 2006). These beneficial effects were reduced when treating mutant islets with an anti-β1-integrin blocking antibody (Nikolova et al., 2006). Endothelial upregulation of hepatocyte growth factor (HGF) in response to increased insulin and VEGFA levels promotes β-cell proliferation (Crawford et al., 1998; Johansson et al., 2006). In addition, endothelial production of the vasoconstrictor endothelin-1 promotes insulin secretion (Gregersen et al., 1996). Furthermore, distinct expression of EC junctional adherence and cell adhesion molecules such as E-cadherin and neuronal cell adhesion molecule (NCAM) has been shown to correlate with β-cell insulin secretion and may contribute to functional β-cell heterogeneity (Domenico et al., 2007; Karaca et al., 2009; Roscioni et al., 2016).

In contrast, TSP-1 functions as a negative regulator of angiogenesis and β-cell proliferation (via activation of transforming growth factor (TGF)-β1, that maintains β-cells in a non-proliferative state) (Crawford et al., 1998; Jiang et al., 2018). However, sustained depletion of TSP-1 impairs β-cell function due to insufficient TGF-β1 activation (Olerud et al., 2008; Olerud et al., 2011).

Islet β-cells exhibit an abundance of VEGFA expression that is required for the formation of the islet-specific microvascular network, specifically promoting the development of fenestrae (Lammert et al., 2003). β-cell-specific inactivation of VEGFA significantly decreased vascularity, and β-cell mass in islets of Rip-Cre;VEGF^fl/fl^ mice (Brissova et al., 2006; Iwashita et al., 2007). These findings were recapitulated by EC-specific knockout of the VEGFA receptor VEGFR2 in Vegfr2^iΔEC^ mice, significantly decreasing the density of islet capillaries, β-cell numbers and insulin production (Chen et al., 2020b). These findings demonstrate a close reciprocal relationship between islet vasculature and endocrine β-cell function (Olerud et al., 2009).

## Aging of the Endocrine System and Endocrine Tissues

Aging represents a major stress factor on cellular function and increases the risk of age-related diseases and mortality. It is a complex facet that remains incompletely understood. In the endocrine system, aging induces endocrine changes that affect overall health, metabolism, fertility, cognition, and cardiovascular risk (Traub and Santoro, 2010; Vitale et al., 2013).

According to the “geroscience hypothesis,” aging is the common major risk factor underlying multiple chronic diseases (Kennedy et al., 2014; Khosla et al., 2020). Therefore, manipulating the fundamental mechanisms of aging may prevent or alleviate these chronic diseases. The mechanisms of aging can be divided into nine, highly interconnected hallmarks, including genomic instability, epigenetic alteration, telomere attrition, exhaustion of stem cells and cellular senescence (López-Otín et al., 2013; Khosla et al., 2020). Senescent cells typically exhibit gene expression changes, loss of proliferative potential and often develop a senescence-associated secretory phenotype (SASP) (Tchkonia et al., 2013). SASP includes excessive production of inflammatory cytokines that affect stem and progenitor cell function, growth factors and vasopressors, that, in turn, induce inflammation and tissue damage (Coppé et al., 2006; Xu et al., 2015; Khosla et al., 2020).

Cellular senescence also impairs mitochondrial function and reduction of oxygen, leading to the excessive formation of reactive oxygen species (ROS). Elevated ROS levels induce oxidative damage and are associated with increased cytokine levels and chronic, subclinical inflammation, further impairing cellular function (Vitale et al., 2013). In the following sections, we will summarize age-related changes in the endocrine system and their known consequences.

### Age-Dependent Changes in Testis

Aging is associated with a decline in testicular function, whereby both mice and humans exhibit decreased serum testosterone levels and spermatogenesis (Chen et al., 1994; Harman et al., 2001). Testosterone is crucial for endothelial function and regulates vasodilation via upregulation of vascular androgen receptors and production of endothelial-derived NO (Chou et al., 1996; Hanke et al., 2001). Multiple studies have found a link between sex steroid hormone deficiency and endothelial dysfunction (Marin et al., 1999; Sader et al., 2003; Hougaku et al., 2006). For instance, castrated rats showed reduced expression and activity of endothelial NOS that was restored upon testosterone treatment (Marin et al., 1999). Furthermore, reduced testosterone levels cause arterial stiffness (Hougaku et al., 2006), impair arterial reactivity and sexual function (Aversa et al., 2011) and increase the risk and severity of cardiovascular disease and mortality (Khaw et al., 2007; Haring et al., 2010; Li L. et al., 2012), suggesting a protective effect of normal testosterone levels against atherosclerosis.

In contrast to testosterone, FSH and LH levels gradually increase with age, further promoting reduced testosterone secretion (Veldhuis et al., 1992). Increased gonadotropin levels may also reflect the reduced secretion of androgen and estrogen from LCs observed in elderly males. In addition, SCs exhibit reduced secretion of inhibin B, indicating an age-related decline in SC function (Tenover et al., 1988). This age-related hypogonadism is associated with decreased muscle mass and strength and bone density to which testosterone treatment has been identified to reverse these effects (Snyder, 2001).

**FIGURE 2:**
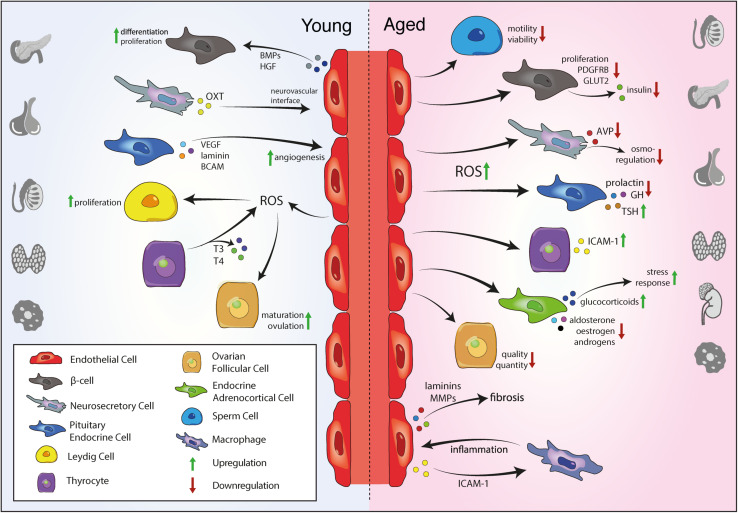
Vascular niche function in the endocrine system during aging. Young ECs secrete angiocrine signals to promote proliferation of endocrine cells and support endocrine function. In the young endocrine system, ECs produce low ROS levels that support Leydig cell proliferation in the testis and promote ovulation OSC maturation in the ovaries. Angiogenic growth factors from pituitary endocrine cells and others promote angiogenesis. Upon aging, endothelial ROS production increases, impairing sperm cell motility, quality and quantity of follicular cells in the ovary and proliferation and hormone production of various endocrine cells, including pancreatic β-cells and endocrine cells and neurosecretory axon terminals in the pituitary gland. In contrast, elevated ROS levels increase the production of inflammatory mediators such ICAM-1 in the thyroid and increases the release of glucocorticoids from the adrenal cortex, promoting the stress response. EC, endothelial cell; ROS, reactive oxygen species; T3, triiodothyronine; T4, thyroxine; VEGF, vascular endothelial growth factor; BCAM, basal cell adhesion molecule; OXT, oxytocin; BMP, bone morphogenetic protein; HGF, hepatocyte growth factor; AVP, arginine vasopressin; GH, growth hormone; TSH, thyroid stimulating hormone; ICAM-1, intercellular adhesion molecule 1; MMP, matrix metalloproteinase.

Moreover, aged testes exhibit increased ROS production by LC mitochondria, inhibiting steroidogenesis (Chen et al., 2001). Low testicular ROS levels have important physiological functions in the testis, contributing to the maintenance of LC proliferation and function and regulating spermatozoa maturation (Griveau and Lannou, 1997; Tai and Ascoli, 2011). However, age-related increase of ROS levels impairs steroidogenesis via the inhibition of steroidogenic enzyme expression and suppression of mitochondrial cholesterol transfer that initiates steroidogenesis (Lee et al., 2009). Furthermore, testicular aging also damages seminiferous tubules and impairs sperm motility and viability and consequently reduces male fertility (Nakamura et al., 2010; Vitale et al., 2013; Figure 2). High ROS levels can result in oxidation of unesterified fatty acids that are very abundant in the cell membrane of spermatozoa, making them highly sensitive to oxidative stress (de Lamirande et al., 1997). Rodent models of aging show increased Ink4a/Arf expression in multiple tissues, including testis and ovaries (Krishnamurthy et al., 2004). The Ink4a/Arf locus encodes the cell cycle inhibitor p16INK4a and can be used as a biomarker of mammalian aging.

### Age-Associated Changes in Ovarian Tissue

During the process of ovarian aging, the pool of oocytes and follicles decreases in quantity and quality (Broekmans et al., 2009; Figure 2). Since ovarian follicular cells represent an important source of steroid hormones, continuous reduction of follicle numbers with age induces ovarian cycle irregularity and impairs female fertility (Michael and Ramkumar, 2016).

In addition, oocyte maturation worsens with age, while the rate of DNA fragmentation and concomitant apoptotic potential increases (Fujino et al., 1996; Tatone et al., 2006). Morphometric follicle analysis demonstrates aged follicles to precociously enter the growth phase compared to younger follicles (Westergaard et al., 2007). This altered follicular growth may contribute to the qualitative and quantitative decline in ovarian follicles with age (Tatone et al., 2008). Ultimately, the pool of follicles is exhausted, and the menstrual cycle can no longer be sustained during menopause (Faddy et al., 1992). Age-related reduced oocyte quality can result in aneuploidy of embryos, fetal death and miscarriages (Andersen et al., 2000; Pellestor et al., 2003). According to the telomere theory of reproductive senescence, aged oocytes may also be susceptible to telomere shortening due to a decline in telomerase activity, impairing fertility and reproduction (Keefe et al., 2005). Inducing telomere shortening in TR-/- mice lacking telomerase activity disrupts meiosis and embryonic cell cycles and promotes embryonic apoptosis (Liu et al., 2002). In women undergoing *in vitro* fertilization, telomere length in oocytes predicts embryo fragmentation, which functions as a marker for apoptosis (Keefe et al., 2005).

Furthermore, reduced follicle quality and ovarian function during aging are associated with oxidative stress. Multiple studies demonstrate increased ROS levels in granulosa cells and oocytes, concomitant with increased levels of mitochondrial DNA deletions and reduced expression of antioxidant enzymes (Seifer et al., 2002; Tatone et al., 2006; Yamada-Fukunaga et al., 2013). Endogenous ROS are required for oocyte maturation, steroidogenesis and CL function and are produced by immune cells and preovulatory follicles to induce ovulation (Shkolnik et al., 2011). However, age-associated accumulation of cyclically produced ROS may lead to DNA damage, telomere shortening and ovarian aging (Behrman et al., 2001; Liu et al., 2003). In line with this, antioxidants such as melatonin (Zhang et al., 2019), coenzyme Q10 (Ben-Meir et al., 2015), and C-phycocyanin (Li et al., 2016) have an anti-aging effect on murine oocytes by regulating mitochondrial function. They reduce ROS levels, reverse the decline of oocyte quality and quantity and restore fertility during reproductive aging.

The age-related drop of follicle numbers also reduces the production of estrogen and progesterone, causing bone loss, hot flashes and other age-related conditions (Finkelstein et al., 2008; Michael and Ramkumar, 2016). Estrogens are known to have a vasodilative effect and pharmacological inhibition of aromatase impaired flow-mediated vasodilation, demonstrating an important regulatory role for endogenous estrogens in endothelial function (English et al., 2001; Lew et al., 2003). Interestingly, multiple studies demonstrate a protective role for estrogens against oxidative stress and aging. Female rats show significantly lower mitochondrial ROS production than male rats and ovariectomy increased oxidative stress levels to those seen in male rats. This could be prevented by estrogen replacement therapy (Borrás et al., 2003). Similarly, estrogens upregulate the expression of antioxidant enzymes and longevity-associated genes via MAPK and NFkB activation (Jose et al., 2011).

### Aging in Thyroid Gland

In the thyroid gland, aging is associated with a decrease in tissue volume and secretion of thyroid hormones while increasing the prevalence of various thyroid diseases (Mariotti et al., 1993, 1995). In elderly individuals without thyroid disease, TSH secretion and serum levels are increased while T_4_ levels remain unchanged (Bremner et al., 2012) and aged mice show decreased serum thyroid hormone levels (da Costa et al., 2001). These findings suggest an age-associated disruption of negative feedback pathways on the hypothalamus-pituitary-thyroid axis (Jansen et al., 2015). Hypothyroidism and increased TSH levels are associated with cognitive impairments (Hogervorst et al., 2008), depression (Medici et al., 2014) increased fracture risk (Blum et al., 2015), cardiovascular disease and mortality (Rodondi et al., 2010). Surprisingly, several studies demonstrate an association between age-related hypothyroidism and longevity in mice and humans (Ooka and Shinkai, 1986; Atzmon et al., 2009; Corsonello et al., 2010; Rozing et al., 2010). However, the mechanisms underlying this association remain unclear.

Thyrocytes produce large amounts of H_2_O_2_ to synthesize thyroid hormones, that results in thyrocytes being constantly subjected to ROS. Protection of thyrocytes against excessive ROS relies on an antioxidant system that is dysregulated in aging, causing an imbalance between ROS and antioxidants that may damage thyroid morphology and function (Vitale et al., 2013). For instance, thyroid levels of the antioxidant selenium decrease with age and may increase thyroid vulnerability to oxidative stress (Akbaraly et al., 2007; Figure 2). Increased ROS levels and oxidative stress also contributes to the development of thyroid autoimmune diseases through fragmentation of thyroglobulin and increased expression of intercellular adhesion molecule 1 (ICAM-1) by thyrocytes (Duthoit et al., 2001; Burek and Rose, 2008).

### Age-Related Modulations in Pituitary Gland

Pituitary hormone secretion exhibits complex and heterogeneous changes during aging (van den Beld et al., 2018; Figure 2). Aging is associated with a progressive decline in pituitary function, due to endocrine deficiency, that, in turn, may contribute to senescence (Vitale et al., 2013). Furthermore, aged pituitary glands show an accumulation of oxidative products that further contributes to aging (Kondo et al., 2001). Increased levels of radicals are also found in the hypothalamus of aging rats, concomitant with reduced antioxidant capacity as measured by glutathione peroxidase activity (Rodrigues Siqueira et al., 2005). These findings suggest an imbalance of oxidant production and antioxidant protection that may cause oxidative damage to cells of the HP axis (Rodrigues Siqueira et al., 2005; Vitale et al., 2013). In addition, aging increases apoptosis of endocrine cells in the adenohypophysis, contributing to impaired hormone production (Nessi et al., 1995).

For instance, pituitary aging decreases GH pulses, causing GH deficiency and decreasing insulin-like growth factor 1 (IGF-1) production (Aimaretti et al., 2003). GH secretion during exercise, sleep and fasting declines with age, particularly in company with abdominal visceral adiposity (Broglio et al., 2003; Veldhuis et al., 2011; Veldhuis, 2013). Moreover, GH activity is positively associated with age-related dysfunction of white adipose tissue and senescent cell accumulation (Stout et al., 2014). Aged GH-deficient and GH-resistant mice with homozygous deletion of the GH receptor (GHR-/-) exhibit increased levels of circulating GH, reduced age-related lipid redistribution and senescent cell burden.

Prolaction secretion in pituitary mammotrophs becomes more irregular in aged men (Kok et al., 2004; Roelfsema et al., 2012).

In contrast, pituitary TSH secretion increases with age and multiple population-based cohort studies demonstrate an association of increased TSH levels with longevity (Atzmon et al., 2009; Rozing et al., 2010; Bremner et al., 2012; Waring et al., 2012). However, confounding factor such as decreased exercise, reduced caloric intake or glucocorticoid exposure may suppress TSH levels during aging (Veldhuis, 2013).

Similar to TSH levels, gonadotropin levels gradually increase in aging men (FSH and LH) and premenopausal women (FSH) (Tenover et al., 1988; Sowers et al., 2008). Aging is also linked to more acidic and sialylated FSH isoforms with a higher half-life and lower *in vitro* bioactivity (Loreti et al., 2009). High FSH levels have been shown to promote osteoclast differentiation and bone resorption in mice (Sun et al., 2006). Genetic deletion of the FSHβ subunit (FSHβ-/- mice) or FSH receptor (FSHR-/- mice) protects against bone loss despite severe hypogonadism, demonstrating a contribution of FSH in hypogonadal bone loss (Iqbal et al., 2006; Sun et al., 2006).

The effects of aging on the stress-responsive HPA-axis remain controversial. Unstressed mean levels of ACTH are reported to remain unchanged, decrease or increase with age (Veldhuis, 2013). These discrepant findings may be indicative of the presence of confounding factors such as alterations in intra-abdominal adipose tissue. Adipose tissue has been shown to increase plasma ACTH levels and impair adrenal sensitivity to ACTH (Ferdinand et al., 2012). In the neurohypophysis, arginine vasopressin (AVP) secretion increases with age (Terwel et al., 1992; Greenwood et al., 2018). Systemically AVP triggers water reabsorption in the kidneys. Therefore, age-related changes in AVP secretion disrupt osmoregulation and water homeostasis (Veldhuis, 2013). Moreover, aging downregulates renal expression of the AVP receptor V2 and the water channel aquaporin 2 in Fischer 344 and Brown-Norway F1 hybrid rats, impairing renal concentrating ability (Tian et al., 2004).

### Age-Dependent Changes in Adrenal Gland

Aging of the adrenal gland is associated with a gradual, sustained increase in glucocorticoids (Yiallouris et al., 2019; Figure 2). This age-related glucocorticoid overload may be caused by increased hippocampal oxidative stress and decreased negative feedback control over the HPA axis that is regulated by glucocorticoid and mineralocorticoid receptors on the hippocampus, hypothalamus and pituitary gland (Kobayashi et al., 2009; Vitale et al., 2013). According to the ‘glucocorticoid vulnerability hypothesis’, these age-related disruptions of the HPA-axis lead to long-term exposure to increased glucocorticoid levels that subsequently causes cognitive impairments (Cheryl, 2008), perhaps contributing to the development of age-related neurodegenerative diseases (Yiallouris et al., 2019). Chronically enhanced glucocorticoid levels also delay and impair the recovery from stressful stimuli in aging (Sapolsky et al., 1983; Lorens et al., 1990; Segar et al., 2009). Moreover, aged adrenals exhibit reduced efficiency of the antioxidant defence system, that may further enhance oxidative damage and senescence (Azhar et al., 1995).

In contrast, other adrenocortical hormones such as aldosterone and the precursor of estrogens and androgens, DHEA, progressively decrease during aging (Hegstad et al., 1983; Orentreich et al., 1984; Labrie et al., 1997) and this decrease is linked to an increased risk in the development of cardiovascular mortality and mental health impairments (Yiallouris et al., 2019). Decreased aldosterone levels are associated with reduced renin activity (Hegstad et al., 1983; Yiallouris et al., 2019). However, the mechanisms underlying these decreases remain unclear. Aging also reduces adrenal androgen production and steroidogenesis. Excessive adrenal ROS levels may cause increased lipid peroxidation and subsequent oxidative damage of cell membranes, particularly in steroidogenic cells that contain high levels of lipids (Azhar et al., 1995; Traub and Santoro, 2010).

### Age-Dependent Changes in Pancreatic Tissue

The pancreas shows an age-related decline of endocrine function that leads to an impairment in glucose homeostasis and metabolism. Aging impairs islet β-cell function and insulin secretion (Figure 2), while simultaneously increasing insulin resistance (Chen et al., 1985; Christina et al., 2009) and the incidence of type 2 diabetes (DeFronzo, 1981). The age-dependent decline in insulin secretion is, in part, caused by a decrease of β-cell sensitivity to incretin stimulation (Chang and Halter, 2003), loss of Sirt1-mediated glucose stimulated insulin secretion (Ramsey et al., 2008), decreased expression of β-cell glucose transporter 2 (GLUT2) (Ihm et al., 2007), decreased mitochondrial function and increased oxidative stress (Cooksey et al., 2004).

Chronically increased ROS levels contribute to decreased proliferation and regeneration and increased apoptosis of β-cells and failure in β-cell function (Maedler et al., 2006; Gu et al., 2012; Vitale et al., 2013). Pancreatic β-cells exhibit a low antioxidant defense capacity, rendering them highly sensitive to oxidative stress (Rashidi et al., 2009). In addition, aging decreases the activity of antioxidant enzymes (e.g., total superoxide dismutase, CuZn superoxide dismutase and glutathione peroxidase), further increasing the ROS burden (Gu et al., 2012). Moreover, aging reduces β-cell levels of PDGFR. PDGFR signaling promotes age-dependent β-cell proliferation via Erk1/2 phosphorylation and activation of the histone methyltransferase Ezh2. Ezh2 levels are decreased in aged β-cells, impairing β-cell replication (Chen et al., 2011). In line with this, conditional Cre-mediated *Pdgfra* knockout (RIP-Cre; Pdgfra^fl/fl^ mice) prevented β-cell expansion and regeneration, while targeted activation of human PDGFRα in β-cells (RIP-Cre; R26-PDGFRA^D842V^) stimulates Erk1/2 phosphorylation and promotes Ezh2-mediated β-cell expansion (Chen et al., 2011).

## Vascular Perturbations During Aging of the Endocrine System

Aging represents a major stress factor for the tissue microenvironment, impairing vascular morphology and function. Vascular aging and its consequences have been extensively studied in the bone marrow microenvironment, demonstrating impaired angiogenesis, vascular integrity and HSC niche function (Kusumbe et al., 2014; Poulos et al., 2017; Singh et al., 2019). In contrast, vascular aging of the endocrine system remains poorly understood. Defining age-related vascular changes in the endocrine system is important to understand mechanisms that drive aging. This may facilitate the rejuvenation of endocrine tissue by manipulation of the vasculature (Almaça et al., 2014).

Vascular aging in the endocrine system is associated with inflammation and fibrosis (Figure 2). For instance, aged pancreatic islet vasculature exhibits increased macrophage density and upregulated expression of inflammatory markers such as ICAM-1 (Almaça et al., 2014). These findings are supported by a recent deep imaging study, revealing increased numbers of perivascular macrophages and fibroblasts in aged endocrine glands (Chen et al., 2020b). Aged pancreatic islets also contain more laminin and exhibit accumulation of fibrotic material in the ECM of islet vasculature (Chen et al., 2020b). In addition, aging increases the expression of MMP genes that are involved in ECM remodeling and fibrosis (Almaça et al., 2014). These findings demonstrate that aging causes inflammation and fibrosis of islet vasculature, threatening islet function. Interestingly, transplantation of aged pancreatic islets into the eye of young mice with diabetes lead to graft revascularization with healthy vessels, islet cell proliferation and restoration of glucose tolerance (Almaça et al., 2014), suggesting vascular aging as a driving force in the age-related decline of pancreas function.

Using deep imaging of endocrine glands and 3D spatial proteomic data, a recent study demonstrates various age-related vascular changes in the endocrine system (Chen et al., 2020b). Aging decreases arterial numbers and microvascular density in pancreas, testis and thyroid in mice and humans. This is accommodated by an abundance of hypoxic regions. Through increasing gap junctions, aging specifically leads to a decline of an islet capillary subpopulation involved in β-cell maintenance and pancreatic angiogenesis. The decline of this subpopulation correlates with a decline in β-cell proliferation during aging. Reactivation of this subpopulation restores β-cell numbers and self-renewal (Chen et al., 2020b).

Furthermore, aging reduces ovarian vascularization and perifollicular blood flow as measured by power doppler ultrasound assessment of aged ovaries (Ng et al., 2004; Costello et al., 2006). This decline of ovarian vascularity results in a reduced supply of oxygen, nutrients and signaling molecules (Tatone et al., 2008; Li Q. et al., 2012). Regulation of follicular development and oocyte quality relies on adequate vascular supply of nutrients and signals mainly provided by perifollicular vascularization (Li Q. et al., 2012). Consequently, reduced oxygen supply is associated with an aged oocyte phenotype and decreased fertilization and developmental potential of oocytes (Van Blerkom et al., 1997; Huey et al., 1999). Aged ovaries also show upregulated VEGF levels likely as an attempt to compensate for hypoxia (Friedman et al., 1997; Klein et al., 2000; Tatone et al., 2008; Fujii and Nakayama, 2010).

Similar to ovarian aging, aged testis exhibit reduced blood flow and perfusion rate. These changes are accompanied by alterations in arterial resistance and microvascular structure, including impaired vasoconstriction in response to noradrenaline and collapse of peritubular capillary networks (Takizawa and Hatakeyama, 1978; Dominguez et al., 2011). In line with this, testicular microvascular oxygen pressure decreases with age. Oxygen transport from testicular microvasculature to the interstitium requires a certain pressure gradient for diffusion. Therefore, this age-associated decline of microvascular oxygen may limit diffusional O_2_ transport from microvessels to testicular mitochondria and hypoxic regions, thereby impairing testicular function (Dominguez et al., 2011).

## Vascular Dysregulation During Endocrine Disorders

Despite altering endocrine function and vasculature, aging also constitutes a major risk factor for endocrine disorders such as diabetes, osteoporosis and vascular disease (Khosla et al., 2020). Diabetes mellitus is one of the most commonly diagnosed endocrine disorders. It describes a group of chronic metabolic disorders characterized by persistent high blood sugar levels (hyperglycemia) caused by insulin resistance, inadequate secretion of insulin or excessive secretion of glucagon (Lipscombe and Hux, 2007; Blair, 2016). Three-dimensional analysis of the pancreas vasculature demonstrated reduced islet vasculature and vascular branch points in nonobese diabetic (NOD) mice compared to wild-type mice. In addition, NOD mice show reduced numbers of islets and β-cell mass, suggesting a crucial role of the complex inter-islet vascular network to maintain islet function and hormone transport (El-Gohary et al., 2012).

Furthermore, diabetes is associated with many comorbidities and vascular complications that are considered the leading cause of morbidity and mortality. These vascular complications include atherosclerosis, hypertension, cardiovascular disease and endothelial dysfunction (Domingueti et al., 2016). Platelets of diabetic patients show increased aggregation and adhesiveness. This platelet hyperactivity triggers and promotes atherosclerosis (Tschoepe et al., 1990, 1995; Yngen et al., 2004). In the arterial vasculature, MMP-mediated degradation of ECM proteins is downregulated, which increases ECM disposition and leads to pathological vascular remodeling (Portik-Dobos et al., 2002). Endothelial dysfunction is linked to increased vascular arginase expression and activity and reduced endothelial production of vasodilating NO. Arginase competes with endothelial NO synthase (eNOS) for its substrate arginine. This reduces arginine availability to eNOS, leading to decreased NO production and impaired vasorelaxation. Instead, superoxide production increases, inducing oxidative stress measured by elevated levels of lipid peroxidation (Tawfik et al., 2006; Romero Maritza et al., 2008).

Insulin resistance, a hallmark of type 2 diabetes, is associated with obesity. Insulin resistance and obesity interact in a complex system and induce a range of metabolic and proinflammatory changes that impair vascular function and structure, increasing the risk of vascular complications (Tounian et al., 2001; Ho et al., 2011; DeMarco et al., 2015; Camastra et al., 2017; Petrie et al., 2018). Activation of the cell-cycle regulator and tumor suppressor protein p53 in adipose tissue crucially contributes to insulin resistance and is linked to obesity.

In Ay mice, ectopic expression of agouti peptide induces excessive calorie intake via disruption of the melanocortin pathway, inducing senescence-like changes in adipose tissue including an accumulation of oxidative stress increased inflammatory cytokine production and activity of senescence-associated beta-galactosidase (Minamino et al., 2009). A similar study with C57BL6/J mice on a high-fat diet supports these findings, demonstrating increased DNA oxidation, DNA damage, reduced telomere length and increased p53 pathway activation in adipocytes (Vergoni et al., 2016). Targeted inhibition of p53 in adipose tissue in Trp53^loxP/loxP^ Fabp4-Cre mice reduces inflammatory cytokine production and improves insulin resistance, while pharmacological activation of p53 stimulates lipolysis and reduces insulin-induced transport of glucose, thereby enhancing inflammation and inducing insulin resistance (Minamino et al., 2009; Vergoni et al., 2016).

A recent study by Avram and colleagues developed a digital biomarker for type 2 diabetes using smartphone-measured photoplethysmography (PPG), that measures heart rate and peripheral blood oxygen saturation (Avram et al., 2020). Here, they developed a deep neural network that analyses smartphone-measured PPG recordings to predict type 2 diabetes development independent of other comorbidities.

Central diabetes insipidus (CDI) describes a deficiency of the hormone AVP, leading to excessive thirst and production of dilute urine. CDI is often caused by degeneration of hypothalamic neurons and is associated with reduced local arterial blood flow and abnormal blood supply to the posterior lobe of the pituitary gland (Maghnie et al., 2004).

Besides diabetes, polycystic ovarian syndrome (PCOS) is considered one of the most prevalent endocrine disorders and is characterized by hyperandrogenism, oligomenorrhea or amenorrhea and ovarian cysts. PCOS is often accommodated by comorbidities such as cardiovascular disease, type-2 diabetes and infertility (Mariana Di et al., 2018). Ovaries of women with PCOS exhibit multiple vascular anomalies that affect follicular blood supply, including increased VEGF levels, blood flow rate and stromal vascularization (Agrawal et al., 1998; Abd El Aal et al., 2005; Alcázar and Kudla, 2012). Ultrasound assessment of ovarian morphology and blood flow in PCOS patients revealed enlarged ovarian size that correlated with increased insulin levels (Carmina et al., 2005). Moreover, increased ovarian blood flow in PCOS patients correlated with elevated levels of testosterone, estradiol and VEGF (Agrawal et al., 1998; Carmina et al., 2005). Increased TGFβ levels and bioavailability may facilitate ovarian angiogenesis and fibrosis in PCOS (Tal et al., 2013; Liu et al., 2015). Furthermore, PDGF-β levels are reportedly decreased in PCOS (Scotti et al., 2014; Di Pietro et al., 2015). Besides stimulating angiogenesis, PDGFRβ signaling is involved in regulating early folliculogenesis (Pinkas et al., 2008). Therefore, decreased ovarian PDGF-β levels may contribute to deregulated angiogenesis and abnormal accumulation of primordial follicles (Scotti et al., 2014).

Cushing’s disease describes the overproduction of glucocorticoids caused by ACTH-secreting pituitary tumors. Immunohistochemical studies revealed a decreased microvascular density and increased vessel diameter in pituitary adenomas (Turner et al., 2000; Takano et al., 2014). Glucocorticoids are known to inhibit angiogenesis and increase TSP-1 levels (Logie et al., 2010; Yang et al., 2018), suggesting that ACTH-secreting tumors may alter vascular architecture. Cushing’s disease is associated with hypercorticism, which is a major cause of glucocorticoid-induced osteoporosis (GIO) (Bolanowski et al., 2015). GIO, in turn, is associated with additional vascular changes such as a decline of PDGF-β levels and bone-specific type H vessels, impairing both angiogenesis and osteogenesis (Yang et al., 2018).

During a process termed endothelial-mesenchymal transition (EndMT), ECs are able to acquire a myofibroblastic or mesenchymal phenotype that includes a loss of cell-cell junctions and the acquisition of migratory and invasive properties (Lin et al., 2012). ECs lose their endothelial markers and express mesenchymal markers such as fibroblast-specific protein 1 (FSP1) and α-smooth muscle actin. This mesenchymal phenotype also includes a loss of cell-cell junctions and the acquisition of migratory and invasive properties. This type of transdifferentiation causes multiple morphological changes and contributes to many pathological processes and diseases, including fibrosis and tumor progression (Zeisberg et al., 2007b; Lin et al., 2012). Using lineage tracing experiments, ECs have been found to undergo EndMT and contribute to the progression of cardiac fibrosis (Zeisberg et al., 2007b). This process is mediated by TGF-β1 and Smad3-signaling that stimulates endothelial proliferation and is able to induce the acquisition of a fibroblast-like phenotype (Zeisberg et al.,2007a,b). In streptozotocin-induced diabetic mice, ECs undergo EndMT and begin to express the fibroblast marker endothelin-1 (ET-1) which itself promotes cardiac fibrosis and heart failure via EndMT-associated fibroblast accumulation (Widyantoro et al., 2010). EndMT has also been identified as a mechanism in the early development of renal fibrosis in diabetic mice. Genetic lineage tracing demonstrated a significant proportion of renal myofibroblasts of endothelial origin in streptozotocin-induced diabetic Tie2-Cre;LoxP-EGFP mice (Li et al., 2009). Furthermore, there is evidence for EndMT as a source of cancer-associated fibroblasts that play an important role in tumor progression and affect the tumor microenvironment through the secretion of ECM molecules and paracrine factors (Lin et al., 2012). In tumors of Tie2-cre; R26Rosa-lox-Stop-lox-LacZ mice, that labels all EC-derived cells with LacZ (β-galactosidase), a portion of FSP1+ and αSMA+ cells were LacZ positive, indicating an endothelial origin of these fibroblasts (Zeisberg et al., 2007a).

## Bone: Vascular Heterogeneity, Aging, and Endocrine Functions

The vasculature of the skeletal system is crucial for delivering nutrients and oxygen to the stem and progenitor cells that reside in specialized vascular niches in the bone marrow (BM). Moreover, BM ECs secrete a variety of angiocrine signals to maintain resident stem cells and regulate bone angiogenesis, osteogenesis and hematopoiesis (Colmone and Sipkins, 2008; Sivan et al., 2019; Stucker et al., 2020). BM ECs show a remarkable heterogeneity based on the distinct expression pattern of vascular cell surface markers such as CD31 (Pecam-1), Endomucin and E-selectin (Winkler et al., 2012; Kusumbe et al., 2014). The most abundant vessels in the BM are fenestrated sinusoidal type L vessels that express low Endomucin and CD31 levels (Kusumbe et al., 2014). Type H endothelium, a vessel subtype that is mainly found in metaphyseal BM near the growth plate, is characterized by high expression of these two markers. Type H vessels are directly connected to arterioles and contain higher oxygen levels and blood flow rate than type L vessels, thereby creating distinct vascular niches in the BM (Kusumbe et al., 2014; Duarte et al., 2018). BM ECs are associated with distinct perivascular cell types that contribute to specialized vascular niches for hematopoietic stem cells (HSCs). Arterioles and type H vessels are supported by pericytes that express PDGFRβ and NG2, whereas sinusoidal type L endothelium is surrounded by CXCL12-abundant reticular (CAR) cells and perivascular LepR+ cells (Sugiyama et al., 2006; Ding et al., 2012; Kunisaki et al., 2013). Furthermore, type H endothelium is associated with osteoprogenitors and couples osteogenesis to angiogenesis by expressing osteogenic and angiogenic factors such as VEGF, PDGF-β and HIF-1α (Kusumbe et al., 2014; Romeo et al., 2019; Rumney et al., 2019; Chen et al., 2020a).

Skeletal aging induces significant morphological changes in BM endothelium, including decreased vascular integrity and increased leakiness (Poulos et al., 2017; Stucker et al., 2020). Aged BM exhibits a significant reduction in arteriolar and type H vessels as well as PDGFRβ-expressing pericytes (Kusumbe et al., 2014, 2016; Singh et al., 2019). This age-related decline of type H endothelium is accompanied by a reduction of osteoprogenitors, subsequently resulting in decreased osteogenesis and bone density (Kusumbe et al., 2016). Metabolic changes in aged endothelium include increased hypoxia and ROS levels that impair angiogenesis (Poulos et al., 2017). Furthermore, BM aging reduces endothelial expression of CXCL12, SCF, and other signals that are essential for HSC maintenance and homeostasis (Kusumbe et al., 2016; Poulos et al., 2017).

The continuous renewal of the skeletal system suggests that bone homeostasis is interlinked with energy metabolism (Karsenty, 2006). This was demonstrated by the discovery that the skeletal system was capable of secreting a range of hormones that are important for energy metabolism and fertility. Osteocalcin (OCN) secreted from osteoblasts modulates a range of physiological parameters. OCN controls glucose-stimulated insulin secretion and proliferation of β-cells in the pancreas. Moreover, OCN signals to the muscle, live and adipocytes to regulate insulin sensitivity (Lee et al., 2007). OCN secreted from the bone is important in male fertility in mice and humans by increasing testosterone synthesis in LCs (Oury et al., 2011). Osteocalcin regulates murine and human fertility through a pancreas-bone-testis axis (Oury et al., 2013a). Male and female Ocn-null mice show docility and a reduction in spatial learning, which was shown to be due to OCN being able to cross the blood-brain barrier to stimulate hippocampal development and neurotransmitter synthesis (Oury et al., 2013b).

Fibroblast growth factor 23 (FGF23) is produced by osteocytes to regulate 1-alpha-hydroxylase, serum phosphate and para-thyroid hormone (PTH), providing an important signal in phosphate metabolism in the kidney to regulate renal homeostasis (Urakawa et al., 2006; Ben-Dov et al., 2007; Karsenty and Olson, 2016). It has recently been shown that lipocalin (LPN) specifically secreted from osteoblasts regulates food intake in mice (Mosialou et al., 2017). Furthermore, the function of regulation lipocalin is conserved in higher-order primates to regulate hunger (Petropoulou et al., 2020).

## Conclusion

The endocrine system consists of various glands that produce and secrete hormones to regulate a wide range of physiological processes and maintain the homeostasis. As hormone transport takes place via the bloodstream, endocrine glands are vascularized with a dense microvascular network (Hiller-Sturmhöfel and Bartke, 1998). This dense vascularization pattern is crucial for sensing changes in blood composition and transporting hormones and regulatory signals (Katoh, 2003; Jang et al., 2017). Moreover, the microvasculature provides a microenvironment that harbors stem and progenitor cells, regulating their survival, maintenance and differentiation. This vascular niche also interacts with endocrine cells to support and maintain efficient gland function (Ballian and Brunicardi, 2007; Colin et al., 2013).

Aging of the endocrine system significantly alters the vascular network of the endocrine system, decreasing vascular density and function. This vascular decline disrupts the blood and disrupts the tissue microenvironment, amalgamating in impairment of endocrine gland function. Thereby, vascular changes and associated microenvironmental alterations in the aging endocrine system may contribute to tissue aging and may be involved in the pathogenesis of various endocrine disorders.

## Author Contributions

SS wrote the original draft. SS and JD revised the review. AK designed the review structure and edited the manuscript. All authors contributed to the article and approved the submitted version.

## Conflict of Interest

The authors declare that the research was conducted in the absence of any commercial or financial relationships that could be construed as a potential conflict of interest.
